# Brief report: The uricase mutation in humans increases our risk for cancer growth

**DOI:** 10.1186/s40170-021-00268-3

**Published:** 2021-09-15

**Authors:** Mehdi A. Fini, Miguel A. Lanaspa, Eric A. Gaucher, Brian Boutwell, Takahiko Nakagawa, Richard M. Wright, Laura G. Sanchez-Lozada, Peter Andrews, Kurt R. Stenmark, Richard J. Johnson

**Affiliations:** 1 Division of Pulmonary and Critical Care Medicine, University of Colorado Anschutz Medical Center, RC2, Room 8120, Mail stop B-133, 12700 East 19th Avenue, Aurora, CO 80045 USA; 2 Division of Renal Diseases and Hypertension, University of Colorado Anschutz Medical Center, Aurora, CO USA; 3 Department of Biology, Georgia State University, Atlanta, GA USA; 4 The University of Mississippi School of Applied Sciences and the John D. Bower School of Population Health, Jackson, MI USA; 5 Department of Nephrology, Rakuwakai Otowa Hospital, Kyoto, Japan; 6 Department of Cardio-Renal Physiopathology, INC Ignacio Chávez, Mexico City, Mexico; 7 Department of Earth Sciences, Natural History Museum, London, UK; 8 Department of Pediatrics, University of Colorado Anschutz Medical Center, Aurora, CO USA; 9 Rocky Mountain VA Medical Center, Aurora, CO USA

**Keywords:** Obesity, Tumor growth, Fructose, Uric acid, Uricase, Thrifty gene

## Abstract

**Background:**

Recent studies suggest that fructose, as well as its metabolite, uric acid, have been associated with increased risk for both cancer incidence and growth. Both substances are known to cause oxidative stress to mitochondria and to reduce adenosine triphosphate (ATP) production by blocking aconitase in the Krebs cycle. The uricase mutation that occurred in the Miocene has been reported to increase serum uric acid and to amplify the effects of fructose to stimulate fat accumulation. Here we tested whether the uricase mutation can also stimulate tumor growth.

**Methods:**

Experiments were performed in mice in which uricase was inactivated by either knocking out the gene or by inhibiting uricase with oxonic acid. We also studied mice transgenic for uricase. These mice were injected with breast cancer cells and followed for 4 weeks.

**Results:**

The inhibition or knockout of uricase was associated with a remarkable increase in tumor growth and metastases. In contrast, transgenic uricase mice showed reduced tumor growth.

**Conclusion:**

A loss of uricase increases the risk for tumor growth. Prior studies have shown that the loss of the mutation facilitated the ability of fructose to increase fat which provided a survival advantage for our ancestors that came close to extinction from starvation in the mid Miocene. Today, however, excessive fructose intake is rampant and increasing our risk not only for obesity and metabolic syndrome, but also cancer. Obesity-associated cancer may be due, in part, to a mutation 15 million years ago that acted as a thrifty gene.

Fructose is a major component of table sugar (sucrose) and high fructose corn syrup (HFCS), which are the two most common added sugars in the western diet. Sugar and HFCS are present in over 70% of processed foods and accounts for 15% of the calories in the average diet, and in some individuals, intake is 25% or more. Recent studies suggest that fructose in added sugars is a major risk factor not only for obesity and metabolic syndrome, but also for cancer [1–4]. One potential mechanism by which fructose may increase the risk for cancer is by reducing mitochondrial function while stimulating glycolysis, as tumor cells often live in hypoxic microenvironments [3, 5].

A unique aspect of fructose metabolism is that it results in rapid consumption of adenosine triphosphate (ATP), associated with adenine nucleotide degradation and the formation of uric acid [6]. Our group has found that this pathway is critical for how fructose inhibits mitochondrial function, as the uric acid causes cellular and mitochondrial oxidative stress that interrupts the Krebs cycle by blocking aconitase while also reducing beta fatty acid oxidation via inhibitory effects on enoyl CoA hydratase [7–9]. This is consistent with observations that hyperuricemia is a risk factor for cancer [10] including by meta-analysis of epidemiological studies [11] as well as Mendelian randomization studies [12].

Humans have higher serum uric acid levels compared to most mammals due to a mutation in the uricase gene. Uricase is an enzyme that degrades uric acid, and most mammals that express uricase maintain serum uric acid levels in the 1 to 3 mg/dl range. However, the great apes and humans lost uricase through a series of mutations that progressively reduced activity of uricase until it was completely silenced around 15 million years ago [13]. The consequence was an increase in serum uric acid to the 3 to 4 mg/dl range, which then has subsequently increased with western diets rich in fructose-laden sugars and purines [14]. Today nearly 20 million people in the USA have serum uric acid levels greater than 7 mg/dl that place them at increased risk for cancer, obesity, metabolic syndrome, and gout [1–4].

We have previously studied the uricase mutation and shown that it likely functioned as a thrifty gene [13, 15–17]. Specifically, at the time of the mutation during the mid-Miocene, ancestral apes were living in both Europe and Africa and living primarily on fruits. At that time, there was global cooling with a loss of fruit availability during the cooler season in Europe that caused great nutritional stress. Over a period of several million years, there was progressive starvation and extinction of apes in Europe. However, based on the fossil record, some apes survived and returned to Africa to become the ancestors of African great apes and humans, while others migrated to southeast Asia to become the ancestor of the orangutan [18, 19]. By resurrecting the extinct uricase, we were able to show that its loss in human hepatocytes was associated with enhanced fat accumulation and gluconeogenesis in response to fructose, suggesting the mutation provided survival benefits to ancestral apes [13, 17]. However, in today’s society in which excessive intake of fructose and marked hyperuricemia are occurring, the consequence is the emergence of obesity and diabetes [16].

Here we wished to test whether the uricase mutation might also have unwittingly increased our risk for cancer growth and spread.

## Materials and methods

### Reagents

Most reagents, buffers, substrates, uric acid, uricase, and oxonic acid were purchased from Sigma Chemical Company (St Louis, MO, USA). Media for cell culture were obtained from Gibco/BRL (Bethesda, MD, USA). Hematoxylin Nuclear Counterstain (H-3401) was purchased from Vector Laboratories (Burlingame, CA, USA) for use in hematoxylin and eosin tissue staining.

### Cells and culture conditions

Mouse mammary carcinoma cell line (E0771) were a kind gift from Dr. Mikhail Kolonin, The Brown Foundation Institute of Molecular Medicine, Houston, TX. Mouse mammary carcinoma cell E0771 and cells were grown in RPMI 1640 containing 2 mM l-glutamine, 2 g/l sodium bicarbonate, pH 7.4, 1X of antibiotic/antimycotic solution, 5 μg/ml insulin, and 10% endotoxin free fetal bovine. Cells were maintained at 37 °C in 95% air/5% CO_2_, fed every 2 days, and split 1:3 when at or near confluency.

### E0771 syngeneic breast cancer animal model and pharmacologic inhibition of uricase

Using pharmacologic approach and to inhibit uricase, control female mice (C57BL/6 JAX) were placed on standard mouse chow (Ctr-CHOW, Teklad, Madison WI) or chow containing oxonic acid (OA-CHOW). After 1 month of being fed on the special chow, E0771 cells were implanted into the mammary fat pads. After 28 days, mice were sacrificed. Macroscopic tumor volume was measured and lung histology were analyzed for lung metastasis.

### Transgenic mice

Homozygous uricase knockout mice (UOX KO) were backcrossed with C57BL/6 JAX for 10 generations at Denver altitude. Homozygous mice with lack of uricase activity and heterozygous littermates with normal uricase activity were used for experiments. Uricase overexpressing transgenic mice (ssUOX Tg) were also backcrossed with C57BL/6 JAX for 10 generations at Denver altitude. Transgenic mice with control littermates were used for experiments. Age matched female mice were implanted with E0771 mouse mammary breast cancer cells. Serial tumor volume was measured during a 28-day growth period at days 7, 14, and 28. Primary tumors were harvested and examined histologically. Lungs, kidneys, and heart along with blood serum were recovered and analyzed. All animal procedures were performed in accordance with the guidelines of the Institutional Animal Care and Use Committee of the University Colorado Denver Anschutz Medical Campus.

## Results

### Pharmacologic inhibition of uricase stimulates tumor growth, progression, and lung metastasis

**Fig. 1:**
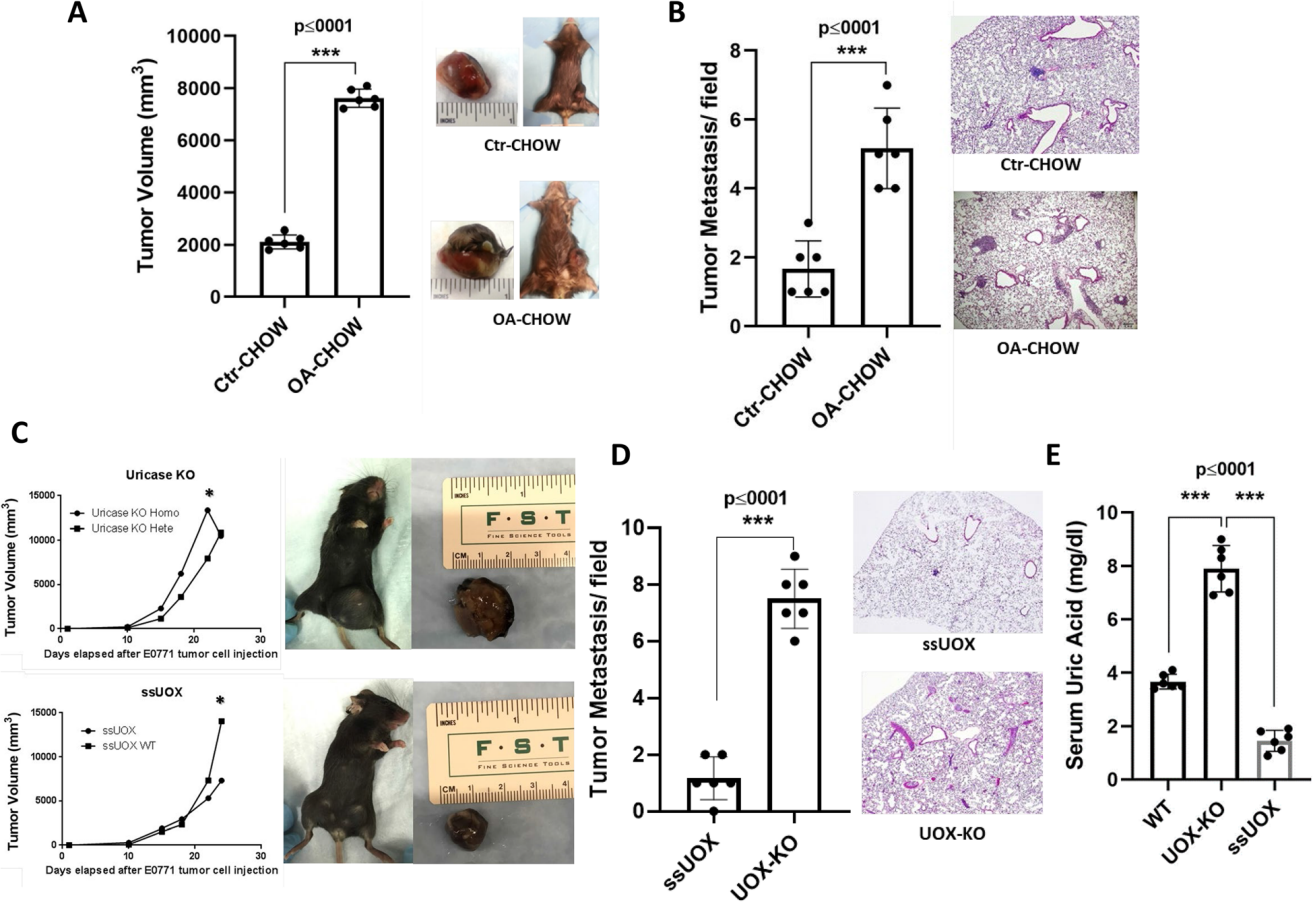
Promotion of tumor growth and metastasis in mice lacking active uricase. Pharmacologic inhibition of uricase augmented the growth and distant metastasis of E0771 syngeneic breast cancer in vivo. Macroscopic tumor volume was measured (**A**) and lung histology were analyzed for lung metastasis (**B**) (tumor volume and lung metastasis shown as histogram, *n*=6 mice per group). Genetic inhibition of uricase using homozygous knockout mice (UOX KO) compared with control as well as mice overexpressing uricase (UOX Tg) were used for experiments. Tumor volume was measured during a 28-day growth period (**C**) (tumor volume shown as histogram, from n=3 independent experiments). At the end of experiment, mice were sacrificed and lung histology were analyzed for lung metastasis (**D**) (tumor metastasis shown as histogram, *n*=6 mice per group). Blood samples were collected at the end of experiment. Serum samples from control non-tumor bearing mice were analyzed for urate level (**E**) (serum uric acid level shown as histogram, *n*=6 mice per group). The threshold for statistically significance is indicated as *p ≤0.05, *** *p* ≤0.001

C57b/6 mice were placed on standard chow (Ctr-CHOW) or chow containing the uricase inhibitor, oxonic acid (OA-CHOW). After 1 month on the different chows, the mice were injected with E0771 breast cancer cells into their mammary fat pads. Four weeks later, mice were sacrificed. Double-blind analysis documented significant increase in primary tumor volume (Fig. 1A) and microscopic lung metastasis (Fig. 1B) in the uricase-inhibited mice compared to control mice.

### Genetic manipulation of uricase modulates tumor growth, progression, and lung metastasis

We also performed similar experiments using homozygous uricase knockout mice (UOX KO) and control littermates. A similar study was performed in which we injected highly aggressive E0771 breast cancer cell lines into the mammary fat pads followed by sacrifice 4 weeks later. Our data show significant augmentation of both primary tumor volume (Fig. 1C) and lung metastases (Fig. 1D) in these mice when compared to their control littermates. Interestingly these were attenuated in transgenic mice overexpressing uricase (ssUOX Tg). Besides genetic confirmation, we determined the serum level of uric acid as a specific determinant of uricase activity which shows high level of serum uric acid in UOX KO vs ssUOX Tg transgenic mice (Fig. 1E).

## Discussion

Here we performed preliminary studies to determine if the loss of uricase could affect cancer growth. Our hypothesis was based on the fact that the uricase mutation has been shown to raise uric acid which not only drives many of the metabolic effects of fructose [1], but also enhances fructose metabolism and production [20, 21]. One of the effects of fructose is to stimulate the Warburg effect, by suppressing mitochondrial function while stimulating glycolysis [3]. This effect results in less oxygen need for the organism. Recently, it was shown that the naked mole rat produces fructose as a mechanism to protect itself from the hypoxia that is present in the underground burrows where it lives [5]. However, while the Warburg effect might be of benefit for animals trying to survive hypoxic conditions, it might also be a mechanism for stimulating cancer growth, for this can allow tumor cells to multiply in tissues with minimal blood supply.

Here we tested the hypothesis using two different approaches. First, we inhibited uricase using oxonic acid and found that this procedure led to a more rapid growth of the breast cancer cells as well as increased metastases. We also showed the same effect when using uricase knockout mice, and a tendency for less tumor growth and metastases in a uricase transgenic mouse. These studies provide tantalizing data that suggest uric acid may have a role in tumor growth.

Supporting this concept has been the observation that hyperuricemia is associated with both obesity and cancer [10], and has been found to increase the risk for cancer incidence and mortality by meta-analysis [11] and Mendelian randomization studies [12]. Furthermore, allopurinol, a xanthine oxidase inhibitor that blocks uric acid formation, reduces breast tumor growth and metastases [22] as well as colonic cancer tumorigenesis [23] in murine models.

In contrast to these latter studies, there is also some evidence that xanthine oxidoreductase may be important for mammary cell differentiation, and that its contribution to the redox balance in tumor cells may alter tumor growth [24]. Thus, further studies are important to determine whether the effects of the uricase mutation to accelerate tumor growth relates to a certain range of uric acid levels and to determine how this relates to the current intake of fructose and levels of serum uric acid in our population.

We would like to suggest that, while the uricase mutation was of benefit by aiding the survival of ancestral hominids, the mutation is maladaptive in current society in which fructose intake is high. Not only is the mutation increasing our risk for obesity and diabetes, but it may also be increasing our risk for cancer. These findings could explain why cancer is increased in subjects with obesity and metabolic syndrome, for both conditions are associated with excessive fructose intake and hyperuricemia. It also suggests that more basic science and clinical studies should be done on the role of both fructose and uric acid in cancer, and also suggests blocking fructose or uric acid metabolism may be an additional target for cancer therapies.

At a deeper level, this study enforces the concept of how evolutionary pressures can lead to changes that were beneficial in one environment yet be deleterious in another. Specifically, cancer growth and metastases may represent another casualty from the loss of uricase, emphasizing the importance of the “thrifty gene” hypothesis in human biology [15].

## Data Availability

The data set in Fig. 1 is available upon request to Dr Mehdi A Fini at Mehdi.Fini@cuanshutz.edu.

## Abbreviations

ATP: Adenosine triphosphate
Ctr-chow: Control chow
HFCS: High fructose corn syrup
OA-Chow: Chow containing oxonic acid
UOX KO: Uricase knockout mice
ssUOX Tg: Uricase overexpressing transgenic mice

## References

[1] Johnson RJ, Nakagawa T, Sanchez-Lozada LG, Shafiu M, Sundaram S, Le M (2013). Sugar, uric acid, and the etiology of diabetes and obesity. Diabetes..

[2] Johnson RJ, Segal MS, Sautin Y, Nakagawa T, Feig DI, Kang DH (2007). Potential role of sugar (fructose) in the epidemic of hypertension, obesity and the metabolic syndrome, diabetes, kidney disease, and cardiovascular disease. Am J Clin Nutr..

[3] Nakagawa T, Lanaspa MA, Millan IS, Fini M, Rivard CJ, Sanchez-Lozada LG, Andres-Hernando A, Tolan DR, Johnson RJ (2020). Fructose contributes to the Warburg effect for cancer growth. Cancer Metab..

[4] Goncalves MD, Lu C, Tutnauer J, Hartman TE, Hwang SK, Murphy CJ, Pauli C, Morris R, Taylor S, Bosch K, Yang S, Wang Y, van Riper J, Lekaye HC, Roper J, Kim Y, Chen Q, Gross SS, Rhee KY, Cantley LC, Yun J (2019). High-fructose corn syrup enhances intestinal tumor growth in mice. Science..

[5] Park TJ, Reznick J, Peterson BL, Blass G, Omerbasic D, Bennett NC (2017). Fructose-driven glycolysis supports anoxia resistance in the naked mole-rat. Science..

[6] Maenpaa PH, Raivio KO, Kekomaki MP (1968). Liver adenine nucleotides: fructose-induced depletion and its effect on protein synthesis. Science..

[7] Lanaspa MA, Cicerchi C, Garcia G, Li N, Roncal-Jimenez CA, Rivard CJ, Hunter B, Andrés-Hernando A, Ishimoto T, Sánchez-Lozada LG, Thomas J, Hodges RS, Mant CT, Johnson RJ (2012). Counteracting roles of AMP deaminase and AMP kinase in the development of fatty liver. PLoS ONE..

[8] Lanaspa MA, Sanchez-Lozada LG, Choi YJ, Cicerchi C, Kanbay M, Roncal-Jimenez CA, Ishimoto T, Li N, Marek G, Duranay M, Schreiner G, Rodriguez-Iturbe B, Nakagawa T, Kang DH, Sautin YY, Johnson RJ (2012). Uric acid induces hepatic steatosis by generation of mitochondrial oxidative stress: potential role in fructose-dependent and -independent fatty liver. J Biol Chem..

[9] Sanchez-Lozada LG, Lanaspa MA, Cristobal-Garcia M, Garcia-Arroyo F, Soto V, Cruz-Robles D (2012). Uric acid-induced endothelial dysfunction is associated with mitochondrial alterations and decreased intracellular ATP concentrations. Nephron Exp Nephrol..

[10] Fini MA, Elias A, Johnson RJ, Wright RM (2012). Contribution of uric acid to cancer risk, recurrence, and mortality. Clin Transl Med..

[11] Yan S, Zhang P, Xu W, Liu Y, Wang B, Jiang T, Hua C, Wang X, Xu D, Sun B (2015). Serum uric acid increases risk of cancer incidence and mortality: a systematic review and meta-analysis. Mediators Inflamm..

[12] Kobylecki CJ, Afzal S, Nordestgaard BG (2017). Plasma urate, cancer incidence, and all-cause mortality: a Mendelian randomization study. Clin Chem..

[13] Kratzer JT, Lanaspa MA, Murphy MN, Cicerchi C, Graves CL, Tipton PA, Ortlund EA, Johnson RJ, Gaucher EA (2014). Evolutionary history and metabolic insights of ancient mammalian uricases. Proc Natl Acad Sci U S A..

[14] Johnson RJ, Titte S, Cade JR, Rideout BA, Oliver WJ (2005). Uric acid, evolution and primitive cultures. Semin Nephrol..

[15] Johnson RJ, Andrews P (2010). Fructose, uricase, and the back-to-Africa hypothesis. Evol Anthropol..

[16] Johnson RJ, Andrews P (2015). The fat gene: a genetic mutation in prehistoric apes may underlie today’s pandemic of obesity and diabetes. Scientific American..

[17] Cicerchi C, Li N, Kratzer J, Garcia G, Roncal-Jimenez CA, Tanabe K, Hunter B, Rivard CJ, Sautin YY, Gaucher EA, Johnson RJ, Lanaspa MA (2014). Uric acid-dependent inhibition of AMP kinase induces hepatic glucose production in diabetes and starvation: evolutionary implications of the uricase loss in hominids. FASEB J..

[18] Begun DR (2000). Middle Miocene hominoid origins. Science..

[19] Andrews P, Kelley J (2007). Middle Miocene dispersals of apes. Folia Primatol (Basel)..

[20] Lanaspa MA, Sanchez-Lozada LG, Cicerchi C, Li N, Roncal-Jimenez CA, Ishimoto T, le M, Garcia GE, Thomas JB, Rivard CJ, Andres-Hernando A, Hunter B, Schreiner G, Rodriguez-Iturbe B, Sautin YY, Johnson RJ (2012). Uric acid stimulates fructokinase and accelerates fructose metabolism in the development of fatty liver. PLoS One..

[21] Sanchez Lozada LG, Andres-Hernando A, Garcia-Arroyo FE, Cicerchi C, Li N, Kuwabara M (2019). Uric acid activates aldose reductase and the polyol pathway for endogenous fructose production and fat accumulation in the development of fatty liver. J Biol Chem..

[22] Oh SH, Choi SY, Choi HJ, Ryu HM, Kim YJ, Jung HY, Cho JH, Kim CD, Park SH, Kwon TH, Kim YL (2019). The emerging role of xanthine oxidase inhibition for suppression of breast cancer cell migration and metastasis associated with hypercholesterolemia. FASEB J..

[23] Kato J, Shirakami Y, Yamaguchi K, Mizutani T, Ideta T, Nakamura H, Ninomiya S, Kubota M, Sakai H, Ibuka T, Tanaka T, Shimizu M (2020). Allopurinol suppresses azoxymethane-induced colorectal tumorigenesis in C57BL/KsJ-db/db mice. Gastrointest Disord.

[24] Fini MA, Monks J, Farabaugh SM, Wright RM (2011). Contribution of xanthine oxidoreductase to mammary epithelial and breast cancer cell differentiation in part modulates inhibitor of differentiation-1. Mol Cancer Res..

